# The Consequences of Our Changing Environment on Life Threatening and Debilitating Fungal Diseases in Humans

**DOI:** 10.3390/jof7050367

**Published:** 2021-05-07

**Authors:** Norman van Rhijn, Michael Bromley

**Affiliations:** Manchester Fungal Infection Group, University of Manchester, Manchester M13 9PL, UK; norman.vanrhijn@manchester.ac.uk

**Keywords:** fungal disease, climate change, global warming, fungal pathogens, *Aspergillus*, *Candida*, *Cryptococcus*, endemic mycoses

## Abstract

Human activities have significantly impacted the environment and are changing our climate in ways that will have major consequences for ourselves, and endanger animal, plant and microbial life on Earth. Rising global temperatures and pollution have been highlighted as potential drivers for increases in infectious diseases. Although infrequently highlighted, fungi are amongst the leading causes of infectious disease mortality, resulting in more than 1.5 million deaths every year. In this review we evaluate the evidence linking anthropomorphic impacts with changing epidemiology of fungal disease. We highlight how the geographic footprint of endemic mycosis has expanded, how populations susceptible to fungal infection and fungal allergy may increase and how climate change may select for pathogenic traits and indirectly contribute to the emergence of drug resistance.

## 1. Orientation

There is no doubt that our climate is changing. Mean global temperatures have risen by 0.8 degrees Celsius since 1975 and are expected to rise by a further 2 to 5 degrees by 2070 [1,2,3] with local fluctuations predicted to be even more dramatic [4,5]. Average sea temperatures are expected to rise in line with global temperature shifts leading to a rise in water levels due to thermal expansion and melting of the ice caps [6]. Overall rainfall levels will decrease however, as the atmosphere can hold more water, rainfall will be more extreme, leading to more flooding [7]. Generally, land areas will become more arid and saline, winds will become more extreme and there are expected to be changes in the levels of ultraviolet radiation reaching the Earth’s surface [8,9,10]. These changes to our environment alongside geopolitical and geoeconomic pressures will require significant adaptation of human behaviour. Although the effects of anthropomorphic activities, especially climate change, have been extensively discussed in the context of displacement and loss of plant and animal species, the impact on microorganisms is rarely discussed [11]. There is a theoretical concern that climate change will affect the frequency, type, morbidity and mortality rates of infectious diseases and these theories are beginning to be backed up by a growing body of evidence. In this review we will explore the potential impact climate change will have on the fungal infections of man, either in their role as primary pathogens of disease or adjuncts to other infections.

## 2. The Ability to Infect Requires Pathogens to Adapt to a Hostile Host Environment

The fungal kingdom emerged 1.5 billion years ago and can be found all across the globe [12,13,14]. Around 120,000 different fungal species have been catalogued; however, there are estimated to be a further 2.1 to 3.7 million different species yet to be identified [15]. Many of these are thought to be located in little explored habitats, particularly in tropical regions and biodiversity hotspots such as The Guiana Shield and neighbouring Amazon basin regions [16,17,18]. Only a few hundred of the catalogued species are known to infect humans [19] and fewer still are considered primary pathogens. The ability of most fungi to infect humans is thought to be as a consequence of the way they have adapted to their ecological niche and hence they are often considered to be accidental pathogens [20]. The key factor that provides a barrier to infection of warm-blooded mammals is their core temperature, as most fungi are unable to adapt to grow at body temperature [21]. This “thermal restriction” has been hypothesised to be one of the major drivers of the evolution of fungal pathogenic potential [22]. The mammalian host is a hostile environment for pathogens for a number of other reasons, which further restrict fungal growth. Hosts can deploy a number of sequestration strategies to limit micronutrient levels, generate highly localised acidic environments, induce oxidative and aerobic stress and release bioactive antimicrobial agents [23,24]. Primary pathogens evade these host responses by avoiding detection, hijacking the host immune systems, countering with their own bioactive agents, generating competing high affinity nutrient sequestration molecules, and rapidly modulating their metabolism to survive and prosper in nutrient- and oxygen-poor environments [25,26,27,28]. Most fungal pathogens however require components of the host’s immune system to fail or become dampened before they can establish an infection. These failures in the immune system can arise from genetic defects or impacts of other primary infections [23].

As climates change, complex and geographically distinct factors will directly affect the health and stability of local ecosystems [29,30]. Temperatures are predicted to rise, so are levels of CO_2_, aridity, salinity, and nutrient availability [31,32,33,34]. This will have an impact directly on the environment of microbiota, changing competitive landscapes in a way that will favour species that have the flexibility to adapt quickly [35]. In the sections below, we highlight where evidence supports the suggestions that human fungal pathogens will have competitive fitness advantages as their local niches expand bringing them into contact with more people. We will also highlight evidence showing that the number of individuals predisposed to fungal infection will increase, due to increases in primary infections, and how climate change may impact the interactions between pathogens and the host immune system [36].

## 3. How Climate Change Could Affect Species Commonly Associated with Infections of Humans

### 3.1. Cryptococcus

Cryptococcosis, is a fungal disease caused by the encapsulated yeast species *Cryptococcus*, and has a broad host range including humans, dogs and marine mammals. Infections in humans are mainly attributed to two species, *Cryptococcus neoformans* and *Cryptococcus gattii* [37]. *Cryptococcus neoformans* has a worldwide distribution and is a relatively common complication of human immunodeficiency virus (HIV) infection. Estimates suggest that there are over 220,000 cases of cryptococcal meningitis in HIV patients annually. Mortality rates in this patient group are high, primarily due to poor diagnosis and availability of antifungal drugs in the developing world, resulting in 180,000 deaths per year [38,39]. It is predicted by 2050, between 11.6 and 16 million additional HIV cases will be attributed to climate change, driven by economic and behavioural changes [40,41]. If a direct linear relationship between HIV cases and *Cryptococcus* cases is maintained this would result in more than 100,000 additional cases of cryptococcosis and if treatment and diagnostic practices in affected areas remain poor, more than 82,000 deaths would follow.

Until recently the geographic distribution of *C. gattii* was thought to be limited to tropical and subtropical areas in South America, Africa, Asia, and Australia. However, it has recently been identified in Mediterranean regions of Europe and an outbreak of cryptococcosis in the Pacific Northwest revealed that *C. gattii* was endemic in areas of the USA [42,43,44,45]. How it arrived in these geographies is unclear, but anthropomorphic factors such as trade in agricultural products (including trees and livestock), human transmission and natural transmission of spores on air currents by migratory birds and even distribution via tsunamis have been suggested [46]. The expansion and successful survival of *C. gattii* in these territories has been attributed directly to changes in ecological niches caused by global warming [46,47]. The northward expansion of the *C. gattii* species complex through the United States into Canada is a real cause for concern, especially as certain molecular types (VGI—*C. gattii sensu stricto* and VGII—*C. deuterogattii*) are able to infect ostensibly immunocompetent individuals [48,49] and it is noteworthy that, compared to other *Cryptococcus* strains, the strains responsible for the Vancouver Island and Pacific Northwest outbreak; VGIIa and VGIIc, respectively, are more virulent in mouse models of cryptococcosis [50,51,52].

Whether increased temperatures or other changes in some environmental niches may select for strains of *Cryptococcus* with enhanced virulence characteristics is a matter for debate and is difficult to prove experimentally. A body of circumstantial evidence has been used to support this concept; however, the link is not that clear cut. Thermal adaptation is directly linked to the biophysical properties of the cell envelope in several organisms (cell membrane, cell wall and capsule) [53,54]. Increased cell and capsule size have also been correlated to increased thermotolerance in *C. gattii sensu stricto* and *C. deuterogatii* species, when compared to other members of the species group; these species are also more pathogenic [55]. The presence of a capsule has clearly been shown to be a key virulence determinant for *Cryptococcus* species and provides protection from phagocytosis and detection by the host’s immune system [56,57] however the link between capsule size, composition and virulence remains unclear. A number of factors make this determination difficult. The capsule is a dynamic structure that can change due to environmental cues. In vitro, the capsule size of a strain does not always reflect the capsule size in a patient [58] meaning that simple correlations between in vitro capsule size and pathogenicity are not necessarily appropriate. In addition, it has been proposed that strains with larger capsules may have a fitness advantage in the lung with smaller capsules linked to dissemination to the brain. Lastly, it has been shown that hyper- and hypo-capsulating strains have reduced virulence in a murine infection model [59], highlighting that the relationship is not straightforward.

There is a stronger case to link increases in virulence, thermal adaptation and protection from UV light via cell wall melanin content. Melanin protects the cell from phagocytosis by macrophages and subsequent reactive oxygen species-mediated cell damage. Melanisation also protects *Cryptococcus* against extreme temperatures and UV radiation [60], hence, it is possible that UV exposure from increased solar radiation as well as increased temperatures could be selecting for strains with increased melanin content in their cell wall and hence increased pathogenicity [61,62,63,64,65]. This hypothesis has been exemplified in *Aspergillus niger* where strains exposed to higher levels of UV contain more melanin, despite originating from geographically similar locations [66]. Following this rationale, other fungi that have melanin in their spores, such as members of the *Aspergillus* genus (see below) or dematiaceous fungi, such as *Exophiala*, *Cladiphialophora* and *Alternaria* may have a fitness advantage as these can withstand high levels of UV radiation, potentially leading to more cases of aspergillosis or phaeohyphomycosis, respectively [61].

### 3.2. Aspergillus

Aspergillosis is a spectrum of disease that consists of allergic, chronic and invasive forms [67]. The global burden of disease is large with over 11 million people thought to be affected by allergic forms, 3 million people with chronic disease, and over 300,000 cases of invasive aspergillosis annually [68].

The impact of air pollution and climate change on allergic disease has been comprehensively reviewed recently [69]. There are a number of factors that are proposed to contribute to increases in allergy, these include an increase in the bioavailability of fungal allergens in the air due to increased growth of fungi as temperatures rise, increases in dispersal caused by storms and intriguingly but rather speculatively, an increase in expression of allergens. It is also fascinating that certain air pollutants can interfere with and enhance the immunogenic potency of *Aspergillus fumigatus* allergens [70]. Several pollutants are also known to cause direct damage to the lung epithelia and the local microbiome that provides an innate barrier to infection. When this barrier is damaged, spores which would ordinarily be cleared before they shed their immunoprotective outer coat [71], have the opportunity to swell and germinate, releasing a payload of allergenic proteins.

Chronic pulmonary aspergillosis is a complication of chronic obstructive pulmonary disease (COPD) and is often seen as a sequel to tuberculosis (TB) [72]. There is conflicting data about the impact of increasing temperature on the frequency of TB. Notifications have been observed to rise with increasing temperatures in some studies, but this response is not uniform [73] with some studies highlighting increased incidence in infection during the winter [74]. Irrespective of this, COPD and TB have been modelled by the WHO to cause over 6 million deaths globally in 2060, up from 2.5 million currently. This increase is primarily driven by reduced air quality, and increased CO_2_ levels [75,76] and will result in an increased population that will be susceptible to chronic aspergillosis.

Resistance to the azole class of antifungals, which are the first line therapeutics for treatment of aspergillosis is of significant concern. Mortality increases from an already high 30–50% in patients infected with a drug sensitive isolate, to near 90% for those infected with a resistant isolate [77]. Azole resistance in some centres in the Netherlands ranges from 15 to 20% [78]. Evolutionarily, drug resistance can be linked to multiple factors, exposure to the drug in question creating selective pressure for resistance, population size, a pool of pre-existing resistance strains and the fitness cost associated with resistance [79]. Resistance can also result as a by-stander effect from other selective pressures. There are theoretical ways in which climate change has the potential to accelerate the emergence of drug resistance in *A. fumigatus* for a number of unique reasons. Azoles target the biosynthesis of ergosterol, a core component of the fungal membrane. Resistance to the azoles is typically driven by increases in ergosterol content, either by mutations in the gene encoding the target of the azoles (lanosterol demethylase) or regulators of the ergosterol biosynthetic pathway [80,81,82]. Changes in the composition of the cell membrane can lead to increased thermotolerance in *Saccharomyces cerevisiae*; however, it is strains with lower ergosterol content that are more thermotolerant [83,84]. Recently it has been shown that a number of azole-resistant strains isolated from patients had mutations in *hmg1*, a gene that encodes HMG-CoA reductase, an enzyme that appears upstream of lanosterol demethylase. Resistance in these strains is linked to a modification of the sterol composition in the cell membrane, including a reduction in ergosterol content [85]. No direct evidence linking thermotolerance and reduced ergosterol biosynthesis has been described in *A. fumigatus* and the azole-resistant Hmg1 mutants were not assessed for fitness at body temperature, although it should be noted that most strains of *A. fumigatus* are already able to withstand high temperatures in excess of 50 °C [86]. Whether strains with reduced ergosterol content would survive well in the environment, or be more or less pathogenic to humans, is also unclear.

Climate change per se will have a drastic impact on the way we farm. It is estimated 40% of land is used for agriculture, and is still increasing. More intense agricultural activities can alter microbial diversity [87]. Environmental factors are known to influence plant pathogens and impact the effectiveness of chemical treatments [88]. This will lead to different pesticide strategies; either using increased concentrations or multiple applications of pesticides [89]. Fungicide usage will change due to climate change as well [90]. As azole resistance is associated with fungicide usage, we may expect increased trends of resistant isolates within the clinic if the use of azoles is expanded [91,92]. Resistant isolates could subsequently be spread by the aforementioned factors that are likely to be affected by climate change, including air currents, human transmission, transport of agricultural commodities (including flower bulbs) and dispersal by migratory birds [93].

Several species of *Aspergillus* are able to cause disease in humans, but *A. fumigatus* is the most common cause of disease. As *A. fumigatus* is able to adapt to high temperature stress better than any other member of the species group [94,95,96] one would predict that in hotter environments *A. fumigatus* would be isolated in patients more than in temperate climates. Indeed, models suggest that continued elevated temperatures lead to replacement of species such as *Aspergillus flavus* by *A. fumigatus* [97,98,99,100]. Paradoxically this does not seem to be the case outside the laboratory. Despite being less thermotolerant than *A. fumigatus*, *A. flavus* is a predominant etiological agent for aspergillosis in Asia, the Middle East and Africa [101]. This highlights that simple lab-based models for thermotolerance do not capture the complex conditions found in the environment. The reasons for the dominance of *A. flavus* in these regions remains elusive, but it is worrying that *A. flavus* is considered to be intrinsically resistant to the salvage therapeutic amphotericin B. As there are only three classes of antifungal used to treat aspergillosis, this could severely restrict treatment options.

Much is left unknown about how *A. fumigatus* will adapt to increases in global temperature and consequently we know little about how these environmental changes will affect the pathogenic potential of this mould.

### 3.3. Mucorales

Mucormycosis is caused by fungi in the order Mucorales, most commonly by *Mucor*, *Rhizopus* and *Lichtheimia* spp. [102,103] and has an estimated annual incidence of around 10,000 patients per year with rates of around 0.1 to 0.3 per 100,000 people, excluding India and Pakistan [68,104]. The rates of infection in India and Pakistan are 100 times higher than this at c. 14 cases per 100,000 individuals, pushing the annual global incidence up to 300,000. One cited reason for the massive disparity in Mucorales infections in this part of the world are the high numbers of individuals with uncontrolled diabetes; however, rates in India (76% of sufferers with uncontrolled diabetes [105]; 77 million sufferers) are not even five times that in the USA (44% uncontrolled; [106]; 31 million sufferers) and are similar to neighboring countries [107]. Therefore it is difficult to discount that other factors are contributing to disease prevalence [108]. Could the local climate be contributing to the high level of infections in this region? There is little evidence to support a link between climate and Mucorales infections other than the fact that these organisms are highly thermotolerant with some species able to grow in temperatures in excess of 45 °C [109,110]. The problem is, there are few ecological studies that have been carried out to compare relative levels of Mucorales and other fungi in soils and the air in different countries and climates, so it is unclear if the abundance of Mucorales in local environments could be contributing to high infection rates. A single centre study has described an association between mucormycosis infection rates and high temperatures/low precipitation however the numbers evaluated are very low [111].

If local environmental conditions are contributing to Mucorales infection rates, it is unclear why countries with climates not dissimilar to India and Pakistan have relatively low levels of infection [104]. The significant efforts of several healthcare workers in these regions have highlighted the problem with Mucorales infections and have driven more effective monitoring and diagnostic programs [104] so incidence in other areas may be under diagnosed. An increase in Mucorales infections would be of serious concern as most mucoraceous fungi are resistant to key antifungals and mortality rates are high (c. 38%) [112].

Less serious but debilitating cutaneous mucormycosis can occur secondary to penetrating trauma [113] and cases have been associated with extreme weather events, such as tornadoes and tsunamis [114,115]. Climate change is affecting the distribution and variability of tornadoes [116,117]. In addition, an increased frequency of tsunamis, earthquakes and volcanic eruptions has been linked to climate change [118]. As sea levels rise, ocean tides and storms become stronger and the effect of El Niño will likely contribute to a further increase of tsunamis [119]. Taken together, there is a potential for an increase of mucormycosis due to climate change and efforts should be undertaken to establish how significant this risk is.

### 3.4. Candida

*Candida* species are a major cause of nosocomial bloodstream infections, being the fourth cause of mortality and morbidity in the intensive care unit (ICU). Annually, 3000–11,000 deaths are associated with candidemia [120]. Technological advancement in medicine has increased patients at risk for *Candida* infections. These infections are increasing as more solid organ transplants per year are performed, immunosuppression becomes prolonged and more patients enter ICU [121]. The main cause of *Candida* infections is *Candida albicans*. However, the epidemiology of *Candida* infections has changed over recent years [122]. In the USA, *Candida glabrata* now accounts for over one third of all *Candida* infections [123]. This is of concern, as some non-*albicans* species are intrinsically resistant to current antifungal therapies [124]. These changes are not directly attributed to climate change, but have been hypothesised to be due to antifungal usage in the clinic [125]. However, *Candida auris*, which has been placed on the CDC antibiotic resistance threats report register, has emerged as a highly drug resistant pathogen and its appearance and spread have been linked to climate change.

*C. auris* was first characterised in 2009, causing otomycosis in a patient in Japan [126]. However, it has been recently shown that imported cases from India were present in Europe as early as 2007 [127]. Soon after, multiple outbreaks of *C. auris* infections were reported [128,129]. This caused the WHO to include *C. auris* as an emerging problem for antimicrobial resistance surveillance programmes [130]. *C. auris* is intrinsically resistant to most of the antifungals currently in clinical use and has a reported mortality rate of 33–72% [131]. Tracing of *C. auris* revealed the potential origin as the Indian subcontinent [132,133]. From here, it appears it spread rapidly, appearing simultaneously in Africa, South America and Asia [132,134,135]. The sudden and swift emergence of *C. auris* has sparked an interest in the evolution of this fungal pathogen. Potentially, a combination of abiotic stress adaptation and biotic predation, in particular by amoeba, has driven the evolution of thermotolerance and halotolerance in this species, resulting in adaptation to different environmental niches and allowing the breaking of the thermal infection barrier of animals with higher core temperatures [136]. These animals, likely to be avian, subsequently distribute the fungus to urban areas where it could infect humans [137,138].

## 4. The Spectre of Newly Arising Fungal Pathogens

Another example of a newly emerging fungal pathogen whose emergence has been traced to the changing environment is *Emergomyces*. The earliest detection of this species was in 1992 and has now been recognised across four continents [139,140]. Similarly, *Sporothrix* species outbreaks have been increasingly reported in Brazil. While from 1987 to 1998, only 13 cases were reported, from 2010 to 2014, 129 cases were reported in humans and between 2012 and 2017, 101 human infections were reported [141,142]. Interestingly, *Sporothrix* clinical isolates are tolerant to high temperatures [143]. Whilst associations have been made with our changing climate, over the same period, our awareness and ability to diagnose atypical fungal infections has increased. Without further directed research, it will remain unclear what contribution climate change is making to the rise in these infections.

Fungal keratitis has been increasing drastically over the last decade. While traditionally considered to be more prevalent in dry environments, the increased use of contact lenses in environments with high abundance of *Fusarium* species has been linked to a spike in keratitis in the USA, with similar trends seen in Egypt and India [144,145,146]. Organisms responsible for keratitis vary worldwide and even minor damage can leave the eye susceptible to fungal infection as the immune response in this area of the body is somewhat limited and the average temperature on the eye surface is around three degrees Celsius lower than the core body temperature in humans. If, as expected, the abundance of spore forming fungi in the environment increases with climate change [147], even organisms not typically associated with invasive infection may become more common in infections of the eye. An increased number of fungal species more typically associated with plant pathogenesis are now seen as causative agents of keratitis [148,149]. Fungal outbreaks caused by emergent and rare species are still being recognised in previously unknown patient cohorts [150,151]. Again further work needs to be undertaken to differentiate improvements in diagnosis from increases in infection rates linked directly to climate change.

## 5. Geographic Spread of Endemic Mycoses

Endemic mycoses are typically caused by a group of dimorphic fungi within the family Onygenaceae. These organisms have a restricted geographical distribution due to their inability to proliferate outside their specialised ecological niches. They are mainly located in North and South America, Africa and parts of Southeast Asia, and include *Coccidioides*, *Paracoccidioides*, *Histoplasma*, *Blastomyces*, *Talaromyes* and the recently identified *Emergomyces*. Infections caused by these fungi are becoming more common and are increasingly reported in “non-endemic” regions [152,153]. Their emergence has been linked to changes in geoclimatic factors and anthropogenic behaviour [154,155].

Coccidioidomycoses (Valley Fever) is primarily a respiratory infection and results from inhalation of spores that have been liberated from the soil. Spore formation is hypothesised to be promoted by spells of precipitation followed by extreme draught, in combination with high temperature [156]. Spores are then aerosolised during dust storms or human activities that disturb the ground [157]. Valley Fever is endemic in the Midwest of the USA, Mexico and parts of Central and South America [152]. In the Midwest, the prevalence of Valley Fever is 6.1/100,000 people, while in more northern states this is 1.1–3.5 [158]. In these patients, dissemination, meningeal infection or severe pneumonia result in 5–10% mortality and require the long-term use of antifungals [159]. Over the last two decades, a 50 and a 213% increase in Valley Fever has been observed in California and Arizona, respectively [160,161,162,163] with the weather, including a particularly warm and dry period in 2016/17, and unusual precipitation rates cited as potential contributory factors in increases in infection rates [157,164,165,166].

The two causative agents of disease are *Coccidioides immitis* and *Coccidioides posadasii*. *C. posadasii* is thought to have emerged from southern Arizona and expanded northwards into central Arizona around 800,000 years ago and southward into Texas and South America around 500,000 years ago, probably facilitated by the movement of infected mammals [167]. *C. immitis* is believed to have diverged c. 370,000 years ago in the San Joaquin Valley region of California [168]. Until recently their geographical distribution was settled; however, there is clear evidence of modern dispersal of *Coccidioides*.

In keeping with the rest of the United States, rates of coccidiomycosis in the central state of Missouri increased significantly from 2004 through to 2013. Although several of these cases were linked to travel to endemic regions, some were thought to be contracted locally, as patients had not travelled outside their local district. Sampling of environments near the patient’s homes, however, were not able to identify evidence of *Coccidiodes* in the environment [169]. In Washington State, however, *C. immitis* has been repeatedly isolated from soil near to a crash site where a patient is likely to have contracted coccidiomycosis. The origins of the strain have been tracked to San Joaquin by genome sequencing [169]. Subsequent cases of Valley Fever have been identified in the area confirming that *Coccidioides* is now endemic in this region [167,170].

The spread of arid environments, with more dust and moisture may cause a more northern spread of coccidioidomycosis, doubling the endemic area [171,172]. The further expansion of arid areas in the Midwest of the USA and a predicted 240% increase in dust storms may spread Valley Fever to previously nonendemic regions [173,174,175]. Furthermore, increased human activity in arid areas and expansion of urban regions has been linked to the incidence of Valley Fever [157]. Epidemiological models predict that by 2030 cases will increase by 12%, and in 2100, cases will increase by 50% [172].

Paracoccidioidomycosis has been recognised by the WHO as a rare tropical disease and is endemic in South America [152,176,177]. This systemic disease is caused by *Paracoccidioides brasiliensis* and to a lesser extent by the recently discovered *Paracoccidioides lutzii* [178]. These saphrophytic fungi grow within the soil and have been associated with humid regions with moderate temperatures in the vicinity of water [179]. When conidia are aerosolised and inhaled, they can cause an infection in immunocompetent people with mortality rates of around 4% [177]. Over time, changes in the prevalence and distribution of this mycosis have been observed, hypothesised to be linked to human migration and climate change [180]. This increase has been seen around the Amazon regions where construction and internal migration have led to changes in epidemiology [181]. Deforestation and soil disruption as well as increased population density have been linked to paracoccidioidomycosis in other regions [182,183]. The incidence of acute/subacute paracoccidioidomycosis is also associated with higher air temperature and humidity in the year prior to diagnosis [184,185]. Additionally, soil moisture seems to affect prevalence [186]. Modelling of environmental variables with infection rates found a strong link to El Niño events [187]. More frequent extremes as well as global spread El Niño events have been linked to climate change and may contribute to the expansion of paracoccidioidomycosis [1,188,189].

The *Blastomyces dermatitidis* complex is endemic to parts of North America and has been reported in Africa and India [152] and there is evidence that infections are becoming more frequent in more northerly areas of the globe. For example, in Canada, blastomycosis has been reported with increasing frequency, from 0.08 cases per 100,000 people 20 years ago to 0.5 cases now [153]. Inhalation of spores, aerosolised by disruption of soil, can cause infection in humans and animals. *B. dermatitidis* can withstand a wide range of temperatures and its abundance has been associated with drought [190]. Higher temperatures and levels of precipitation in prior seasons are indicative of the expected infection rate during May and October, in which the majority of infections occur [191]. Clusters of infections have been associated with periods of changes in precipitation, followed by drought, similar to *Coccidioides* [192,193,194,195]. Extremes in precipitation and drought are predicted to increase in North America, which may lead to drastic changes in prevalence of *B. dermatitidis* [196,197,198,199].

*Histoplasma capsulatum* is the causative agent of histoplasmosis, and causes infections in South and North America and Sub-Saharan Africa [140]. The fungus proliferates within soil areas with bird or bat droppings [200]. Some 90% of people in the river valleys of the United States are exposed to *H. capsulatum* within their lifetime, but it causes infection in less than 1% of people [201]. However, outbreaks have been observed [202,203]. As of 2019, histoplasmosis has been recognised by the CDC as an endemic mycosis in more northern states of the United States of America [204,205]. While no seasonal or temporal trends in incidence were observed, data from 2011–2014 showed a geographical spread to non-endemic regions [153,206]. In Ontario, Canada, no change in cases has been observed between 1990–2015, while in Alberta, Canada a continuous rise (0.05 to 0.25 per 100,000 people) in cases has been observed from 2015, showing a northward expansion of histoplasmosis [153]. Modelling of the suitable environmental conditions (distance to open water, soil pH and land cover type) revealed that climate change has expanded the geographical niche for *H. capsulatum* [207]. Furthermore, as *H. capsulatum* is also found in bird and bats droppings, the climate change-linked behavioural changes of birds and bats will have an impact on the spread of histoplasmosis [208,209,210]. This has been supported by a shift in outbreaks in rural areas to more urban areas, affecting more people [199]. Interestingly, strains of *H. capsulatum* that grow at elevated temperature or those exposed to more light show increased virulence, suggesting that if temperatures and exposure to UV increases as predicted [197,199,211], selective pressures may drive strains to become more pathogenic [212,213].

## 6. Concluding Remarks

**Figure 1:**
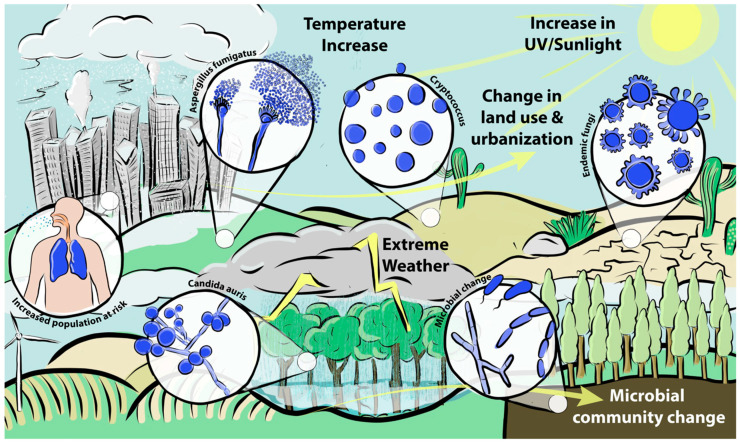
Schematic overview of changes in the epidemiological landscape of fungal pathogens and associated changes in environmental parameters.

Climate change will have an impact on the way we live, farm and interact with our environment. This will change the epidemiological landscape of pathogens, as fungi can readily adapt to changing environments. There is yet much work to do on fungi in relation to climate change, especially since only a small proportion of 1.5 million fungal species have been identified. Our changing environment will likely expose us to fungi with which humans have never interacted. Furthermore, more humans will become at risk for fungal infections. The prevalence and diversity of soil microorganisms will undoubtedly change, due to climate change. This change has already been observed with endemic fungi and with the emergence of novel fungal pathogens (Figure 1). New emerging diseases, such as COVID-19 and severe influenza, are being recognised as risk factors for fungal infections [214,215,216]. The emergence and spread of viral infections is facilitated by increased population density. As climate change drives the increase in urban populations, diseases that predispose to secondary fungal infections are likely to increase [217,218]. However, many questions remain unanswered and more study is required.
